# Supplementary material for: Causal Relationship between Aging and Anorexia Nervosa: A White-Matter-Microstructure-Mediated Mendelian Randomization Analysis
Source: Biomedicines. 2024 Aug 16;12(8):1874. doi: 10.3390/biomedicines12081874 (PMC11351342; doi:10.3390/biomedicines12081874)
Supplement: Supplementary file 1 [file biomedicines-12-01874-s001.zip › Supplementary Figure/Figure S4/Singe SNP plot of MR analysis for TL to ubm-b-1646.pdf]

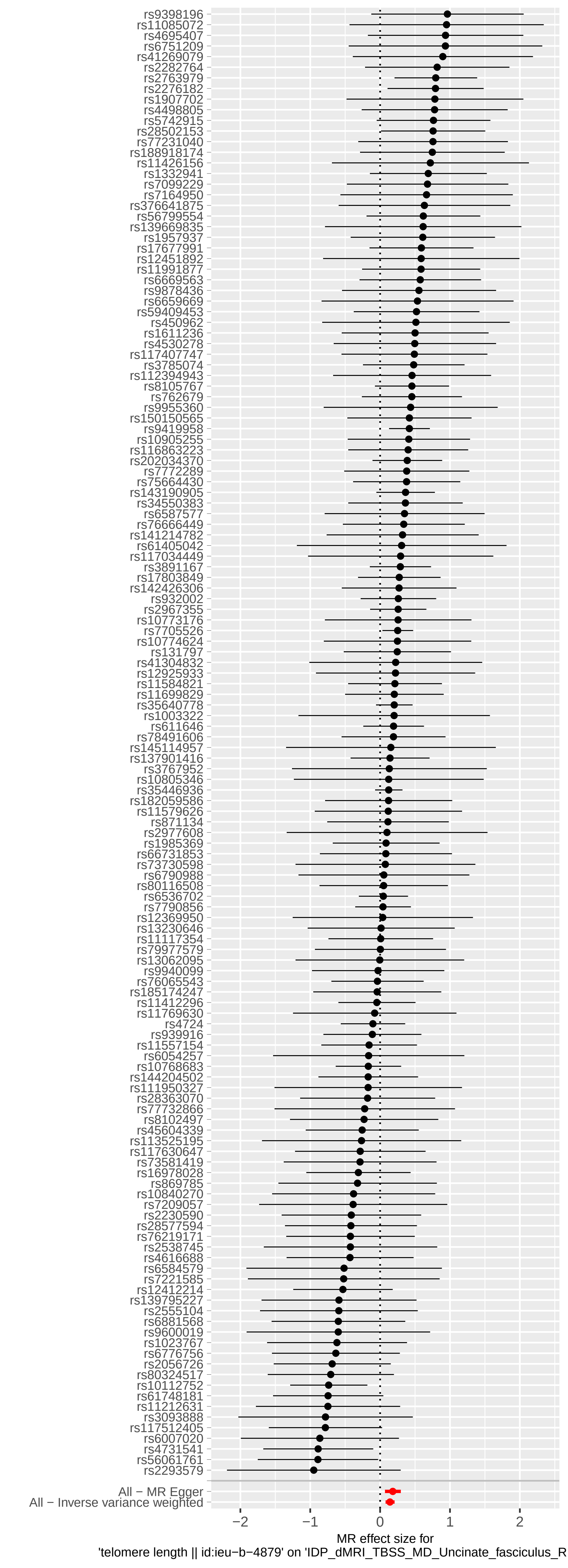
rs9398196
rs11085072
rs4695407
rs6751209
rs41269079
rs2282764
rs2763979
rs2276182
rs1907702
rs4498805
rs5742915
rs28502153
rs77231040
rs188918174
rs11426156
rs1332941
rs7099229
rs7164950
rs376641875
rs56799554
rs139669835
rs1957937
rs17677991
rs12451892
rs11991877
rs6669563
rs9878436
rs6659669
rs59409453
rs450962
rs1611236
rs4530278
rs117407747
rs3785074
rs112394943
rs8105767
rs762679
rs9955360
rs150150565
rs9419958
rs10905255
rs116863223
rs202034370
rs7772289
rs75664430
rs143190905
rs34550383
rs6587577
rs76666449
rs141214782
rs61405042
rs117034449
rs3891167
rs17803849
rs142426306
rs932002
rs2967355
rs10773176
rs7705526
rs10774624
rs131797
rs41304832
rs12925933
rs11584821
rs11699829
rs35640778
rs1003322
rs611646
rs78491606
rs145114957
rs137901416
rs3767952
rs10805346
rs35446936
rs182059586
rs11579626
rs871134
rs2977608
rs1985369
rs66731853
rs73730598
rs6790988
rs80116508
rs6536702
rs7790856
rs12369950
rs13230646
rs11117354
rs79977579
rs13062095
rs9940099
rs76065543
rs185174247
rs11412296
rs11769630
rs4724
rs939916
rs11557154
rs6054257
rs10768683
rs144204502
rs111950327
rs28363070
rs77732866
rs8102497
rs45604339
rs113525195
rs117630647
rs73581419
rs16978028
rs869785
rs10840270
rs7209057
rs2230590
rs28577594
rs76219171
rs2538745
rs4616688
rs6584579
rs7221585
rs12412214
rs139795227
rs2555104
rs6881568
rs9600019
rs1023767
rs6776756
rs2056726
rs80324517
rs10112752
rs61748181
rs11212631
rs3093888
rs117512405
rs6007020
rs4731541
rs56061761
rs2293579
All − MR Egger
All − Inverse variance weighted
−2
−1
0
1
2
MR effect size for
'telomere length || id:ieu−b−4879' on 'IDP_dMRI_TBSS_MD_Uncinate_fasciculus_R
